# Enhancing Myoblast Fusion and Myotube Diameter in Human 3D Skeletal Muscle Constructs by Electromagnetic Stimulation

**DOI:** 10.3389/fbioe.2022.892287

**Published:** 2022-06-22

**Authors:** Lisanne Terrie, Margherita Burattini, Sandra Van Vlierberghe, Lorenzo Fassina, Lieven Thorrez

**Affiliations:** ^1^ Tissue Engineering Lab, Dep. Development and Regeneration, KU Leuven Kulak, Kortrijk, Belgium; ^2^ Dept. of Surgical Sciences, Dentistry and Maternity, University of Verona, Verona, Italy; ^3^ Polymer Chemistry & Biomaterials Group, Centre of Macromolecular Chemistry, Dep. of Organic and Macromolecular Chemistry, Ghent University, Ghent, Belgium; ^4^ Dept. of Electrical, Computer and Biomedical Engineering, University of Pavia, Pavia, Italy

**Keywords:** tissue engineering, skeletal muscle, pulsed electromagnetic field, biophysical stimuli, bioartificial muscle, myotube, human myoblast

## Abstract

Skeletal muscle tissue engineering (SMTE) aims at the *in vitro* generation of 3D skeletal muscle engineered constructs which mimic the native muscle structure and function. Although native skeletal muscle is a highly dynamic tissue, most research approaches still focus on static cell culture methods, while research on stimulation protocols indicates a positive effect, especially on myogenesis. A more mature muscle construct may be needed especially for the potential applications for regenerative medicine purposes, disease or drug disposition models. Most efforts towards dynamic cell or tissue culture methods have been geared towards mechanical or electrical stimulation or a combination of those. In the context of dynamic methods, pulsed electromagnetic field (PEMF) stimulation has been extensively used in bone tissue engineering, but the impact of PEMF on skeletal muscle development is poorly explored. Here, we evaluated the effects of PEMF stimulation on human skeletal muscle cells both in 2D and 3D experiments. First, PEMF was applied on 2D cultures of human myoblasts during differentiation. In 2D, enhanced myogenesis was observed, as evidenced by an increased myotube diameter and fusion index. Second, 2D results were translated towards 3D bioartificial muscles (BAMs). BAMs were subjected to PEMF for varying exposure times, where a 2-h daily stimulation was found to be effective in enhancing 3D myotube formation. Third, applying this protocol for the entire 16-days culture period was compared to a stimulation starting at day 8, once the myotubes were formed. The latter was found to result in significantly higher myotube diameter, fusion index, and increased myosin heavy chain 1 expression. This work shows the potential of electromagnetic stimulation for enhancing myotube formation both in 2D and 3D, warranting its further consideration in dynamic culturing techniques.

## Introduction

Skeletal muscle has a complex yet extremely functional organization. Its contractile function is exerted by aligned myofibers, formed by the fusion of single-nucleated myoblasts, resulting in multinucleated myotubes. Quiescent multipotent stem cells, satellite cells (SCs), located between the basement membrane of the muscle fibers and the sarcolemma (Powell et al., 2002) guarantee the muscle regeneration, when small injuries occur. Consequently, a long-term goal of skeletal muscle tissue engineering (SMTE) is to replicate the structure and function of native skeletal muscle *in vitro* and, to obtain functional constructs. Such an *in vitro* model can enhance research focused on skeletal muscle development and regeneration both in the healthy or diseased states (Vigodarzere and Mantero, 2014; Maffioletti et al., 2018; Khodabukus, 2021). Many approaches for SMTE still focus on static cell culture methods. However, skeletal muscle tissue is highly dynamic, changing its size and power output in response to activity. For this reason, physiological activity is seen as an imperative step to replicate native skeletal muscle structure and function.

This physiological activity can be mimicked *in vitro* through mechanical stimulation or electrical stimulation or the combination of the two (Somers et al., 2017; Maleiner et al., 2018). Several studies have shown how mimicking this physiological activity can advance engineered skeletal muscle. For instance, mechanical loading of 3D cultured C2C12 cells was found to induce significant hypertrophy of the myotubes and augmented maximal force production (Aguilar-Agon et al., 2019). Similarly, repetitive stretch/relaxation cycles were found to increase mean myofiber diameter and myofiber area fraction (%) when applied to 3D cultured human myoblasts (Powell et al., 2002). However, mechanical stimulation does not necessarily stimulate myotube development. For example, stretch was found to negatively influence the maturation of both C2C12 and murine muscle progenitor cells in a 2D and 3D environment (Boonen et al., 2010).

While mechanical stimulation can thus lead to different outcomes, the usage of electrical stimulation in SMTE has led to more consistent improvements in myotube development. Electrical stimulation aims to recapitulate the function of muscle stimulation by fast and slow motor neurons and was found to induce a fast-to-slow shift in engineered muscle when using a stimulation frequency of 10 Hz and an active time of 60% (Khodabukus et al., 2015). An intermittent electrical stimulation at 10 Hz for one week was also found to induce myotube hypertrophy and decrease fatigue resistance and increase force generation (Khodabukus et al., 2019). However, many parameters, such as the period of stimulation and starting point, are not commonly defined and vary among groups. Additionally, results derived from cell lines such as C2C12 are not necessarily translated to primary muscle progenitor cells (Langelaan et al., 2010). In the aforementioned work, 3D constructs engineered with primary progenitor cells were more susceptible to electrical stimuli and showed an overall higher level of maturation than C2C12 constructs.

While mechanical and electrical stimuli have been explored for advancing tissue-engineered skeletal muscle constructs, the use of pulsed electromagnetic field (PEMF) stimulation for SMTE appears to be still unexplored. Nevertheless, the potential of PEMF for tissue engineering has been shown in other fields, especially for osteogenesis and chondrogenesis (Varani et al., 2021). Indeed, PEMF has been demonstrated to promote osteogenesis in nonunion fractures, partly by regulating mesenchymal stem cell (MSC) or osteoblast activity.

Similarly, PEMF stimulation of periodontal ligament stem cells increased the osteogenic potential of the cells (Wang et al., 2017). Moreover, prolonged exposure to PEMF of MSCs isolated from the human umbilical cord was found to enhance the tenogenic potential (Marmotti et al., 2019). In addition, PEMF-based devices are currently sold in the context of muscle pain relief. Still, only a few papers have described the influence of PEMF stimulation on skeletal muscle of patients (Kim et al., 2012; Jeon et al., 2015). Given the scarce evidence of these devices *in vivo* and the lack of studies using these for SMTE, the rationale of this study is to evaluate the value of PEMF stimulation for SMTE by applying PEMF stimulation on both 2D cultured myoblasts and subsequently 3D tissue-engineered skeletal muscle constructs.

## Materials and Methods

### Cell Culture

Human skeletal muscle cells were isolated from a fresh human muscle tissue biopsy, obtained from the Human Body Donation programme of KU Leuven as described previously (Gholobova et al., 2019). The donor provided written consent and ethical committee approval was obtained (NH019-2020-04-02). After isolation, the myoblasts were expanded in gelatin-coated culture flasks with growth medium consisting of DMEM Glutamax (DMEM, Gibco, #41966029), 1% Ultroser solution (Pall Corporation, #15950-017), 10% fetal bovine serum (FBS, Biowest, #S1400-500) and 50 μg/ml gentamicin (Life Technologies, #15750060). Differentiation medium was composed of DMEM Glutamax, 10 ng/ml hEGF (Peprotech, #AF10015), 10 μg/ml insulin (Sigma, #I9278), 50 μg/ml bovine serum albumin (BSA, Sigma, #A2153) and 50 μg/ml gentamicin (Sigma, #15750037) and was used to promote formation of multinucleated myotubes once the cultures reached 80% confluency. 2D cell culture experiments were performed in gelatin-coated 24-well plates using 50,000 cells per well. Cells were subsequently cultured for 2 days in growth medium. Next, the medium was switched to fusion medium to induce myotube formation for 5 days.

### Human Biopsy Characterization: Immunocytochemistry

To determine the percentage of myoblasts, isolated muscle cells were cultured in growth medium in 24-well dishes (5000 cells/well) for 2–3 days until 60–70% confluency. Fixation was performed in a 1:1 methanol–acetone mix at −20°C for 10 min. Next, fixed cells were blocked and permeabilized in blocking buffer containing 1x phosphate buffer saline (PBS), 1% BSA and 0.2% Triton X-100 (Sigma, #X100). Subsequently, cells were incubated overnight at 4°C with a monoclonal mouse antibody against desmin (Sigma, #D1033, 1:200 in blocking buffer). Cells were labelled with a polyclonal rabbit anti-mouse secondary antibody (Alexa Fluor 488, Invitrogen, #A11059) for 30 min in the dark and subsequently incubated with 4′,6-diamidino-2-phenylindole (DAPI, 0.1 μg/ml in PBS, Life Technologies) for 1 h. To determine the fusion index, cells were cultured to 80% confluency in growth medium in 12-well dishes (50,000 cells/well), and then switched to differentiation medium for 4 days to induce fusion into myotubes. Next, myotubes were fixed using 4% formaldehyde, freshly prepared from paraformaldehyde (PFA, Merck, #1040031000) solution weight/volume (w/v), at room temperature (RT) for 10 min followed by methanol fixation at −20°C for 10 min. Next, fixed myotubes were blocked and permeabilized in blocking buffer containing 1x phosphate buffer saline (PBS), 1% BSA and 0.2% Triton X-100 (Sigma, #X100). Subsequently, cells were incubated overnight at 4°C with a monoclonal mouse antibody against tropomyosin (Sigma, T9283, 1:100 in blocking buffer). Myotubes were labelled with a polyclonal rabbit anti-mouse secondary antibody (Alexa Fluor 488, Invitrogen, #A11059) for 30 min in the dark and subsequently incubated with 4′,6-diamidino-2-phenylindole (DAPI, 0.1 μg/ml in PBS, Life Technologies) for 1 h. Images were acquired with Zeiss Zen software by an AxioCam ICc 1 camera mounted on a Zeiss Axiovert 10 microscope. The percentage of myoblasts in a muscle cell population was defined as the ratio of desmin-positive cells to the total amount of cells (identified by the DAPI-stained nuclei). Fusion index was defined as the ratio of tropomyosin-positive cells to the total amount of myoblasts in the population.

### 3D Bio Artificial Muscles

Tissue engineering of the bioartificial muscles (BAMs) was described in detail in previous work (Gholobova et al., 2015; Gholobova et al., 2018; Thorrez et al., 2018; Gholobova et al., 2019; Gholobova et al., 2020). Briefly, myogenic cells were isolated, cultured, and harvested as described. After cell expansion, for each BAM, 2 million cells were mixed with 500 µl of human thrombin (4 U/mL, Stago, #HT1002a). To create the 3D constructs, the cell-thrombin mixture was added to 500 µl of fibrinogen (2 mg/ml, Merck Chemicals, #341576) into 25-mm silicone molds containing two metal pins spaced 20 mm, serving as attachment points. Following a 2-h incubation at 37°C during which the fibrin solidified, BAMs were cultured for the first 2 days in growth medium supplemented with the fibrinolysis inhibitors aprotinin (92.5 μg/ml, Carl Roth, #A1624) and tranexamic acid (400 μM, Sigma, #857653). The medium was switched to differentiation medium with fibrinolysis inhibitors (92.5 μg/ml aprotinin and 400 µM tranexamic acid) from day 3 until day 8 to induce myoblast fusion. After day 8, BAMs were cultured further in growth medium containing fibrinolysis inhibitors until the end of the experiment. The cell culture medium was refreshed every two days.

### Immunohistology of BAMS

For longitudinal analysis of myotube organization, BAMs were washed with PBS and then fixed using 4% PFA solution (w/v) in PBS for 1 h. BAMs were pinned on Styrofoam to preserve their original shape while fixing. Before staining, constructs were additionally fixed in −20°C methanol for 10 min. Fixed constructs were blocked and permeabilized in blocking buffer containing 1x phosphate buffer saline (PBS), 1% BSA and 0.2% Triton X-100 (Sigma, #X100). Subsequently, BAMs were incubated overnight at 4°C with a monoclonal mouse antibody against tropomyosin (Sigma, #T9283, 1:100 in blocking buffer). After extensive washing with PBS, BAMs were incubated with polyclonal rabbit anti-mouse antibody (Alexa Fluor 488, Invitrogen, #A11059, 1:200 or Alexa Fluor 633, Invitrogen, #21,063, 1:200) for 3 h in the dark followed by incubation with DAPI (Life Technologies, 0.1 μg/ml in PBS) for 1 h. BAMs were stored in PBS in the dark until visualized. Images of tropomyosin in 3D BAMs were acquired with Zeiss Zen software by confocal microscopy (Zeiss LSM710) with PlanApochromat 25x/0.8, WD 0.57 mm objective.

For cross-sectional analysis of myotube organization, BAMs were washed with PBS and submerged in optimal cutting temperature compound (OCT) (VWR, #25608-930) and frozen in liquid N_2_-cooled isopentane. Frozen cross-sections (5 µm) were made using a cryostat (Leica CM 1950) and collected on Superfrost Ultra Plus slides (VWR, #631-9483). Sections were placed at room temperature for 5 min followed by washing in PBS to remove OCT. Next, sections were fixed with 4% PFA for 10 min at room temperature. After washing with PBS, sections were permeabilized for 25 min using 1x PBS, 1% BSA and 0.2% Triton X-100. Subsequently, samples were incubated overnight in a moist chamber with rabbit polyclonal anti-laminin (Sigma, #L9393, 1:400 in blocking buffer). Following washing with PBS, sections were incubated for 45 min with a donkey anti-rabbit IgG secondary antibody (Alexa Fluor 488; #A21206; 1:1000 in PBS) in the dark. Lastly, after washing with PBS, slides were mounted with prolong gold antifade mounting medium with DAPI (Thermo Fisher; #P36935). Slides were imaged with an AxioCam ICc 1 camera mounted on a Zeiss Axiovert 10 microscope and images were acquired and stored with Zeiss Zen software. Images were further processed using Fiji ImageJ (version 1.53f51) to determine the cross-sectional diameter of the myotubes. For each biological replicate (*n* = 3), 6 sections were quantified of which 5 pictures were taken for each section.

### Electromagnetic Bioreactor

**FIGURE 1:**
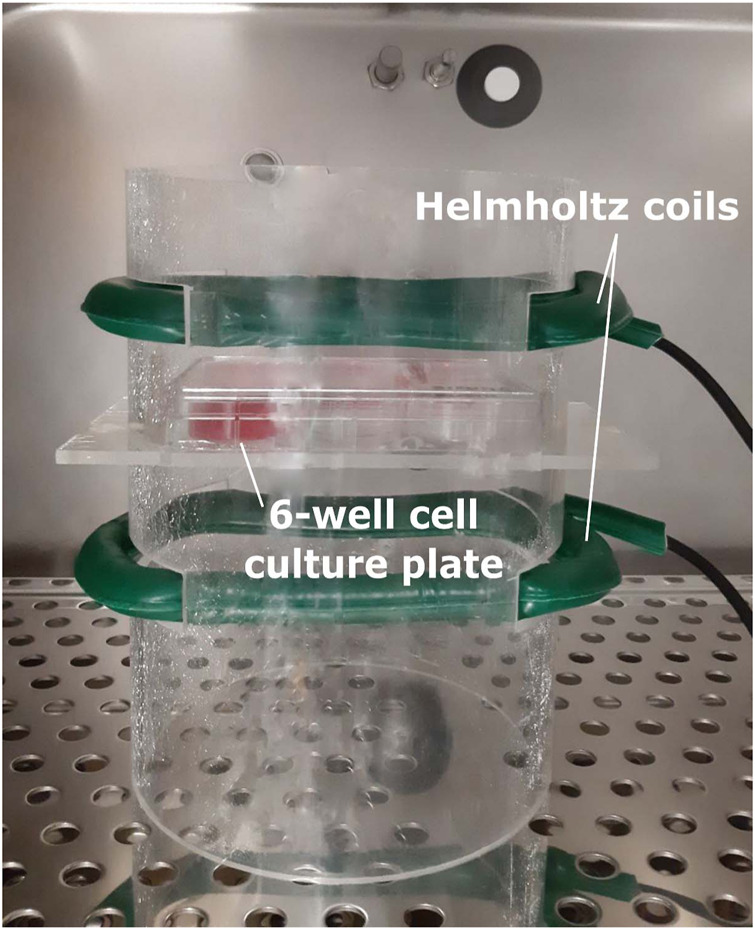
Electromagnetic bioreactor setup with culture plate. The culture plate is located between the Helmholtz coils, which are creating induced currents in the wells.

The electromagnetic bioreactor and its settings were extensively described in previous work (Fassina et al., 2006; Osera et al., 2011; Ceccarelli et al., 2013; Mognaschi et al., 2014). Briefly, it includes a carrying windowed tube of polymethylmethacrylate, in which a culture well plate can be positioned, as represented in Figure 1. Two solenoids (i.e., Helmholtz coils) complete the stimulator, producing a perpendicular magnetic field with respect to the plane of the well plate and inducing a parallel electric field in the wells. In particular, the stimulated cells are 5 cm away from each solenoid plane, and the coils are powered by a Biostim SPT pulse generator (IGEA Medical, Carpi, Italy). Given the position of the solenoids and the characteristics of the pulse generator, the electromagnetic stimulation has the following parameters: intensity of the magnetic field equal to 2 ± 0.2 mT, amplitude of the induced electric tension equal to 5 ± 1 mV, signal frequency of 75 ± 2 Hz, and pulse duration of 1.3 ms. The electromagnetic bioreactor was placed into a standard cell culture incubator at 37°C and exposed to 5% CO_2_.

A more detailed description and analysis of the field distribution and induced current magnitude is provided in Mognaschi et al. (2014). Briefly, the time-varying magnetic induction (with a frequency of 75 Hz) generated a concentric distribution of induced electric currents with a corresponding induced distribution of radial mechanical forces. In other words, stimulated cells are subjected to mechanical vibration at the same frequency as the PEMF. Moreover, the vibration force applied reaches the pN magnitude, which is comparable to the force applied for studying cellular mechanics as reported in Diz-Muñoz et al. (Diz-Muñoz et al., 2010).

### Gene Expression Analysis

To assess differences in the developmental stage of the myotubes, RT-qPCR was performed on the different myosin heavy chain isoforms (MYH1, MYH3, MYH8). We used RPL13A, GAPDH and HSP90AB1 as reference genes. Primers were designed using NCBI/Prime-Blast and verified for an efficiency >90% by a serial dilution analysis. Sequences of the primers are reported in Table 1. As described previously (Gholobova et al., 2020), BAMs were washed in PBS, transferred in freshly prepared lysis buffer (from PureLink™ RNA Mini Kit, Ambon #12183020) containing 1% *β*-mercaptoethanol (Gibco, #31350010) and homogenized on ice using a tip sonicator until the solution appears homogenous (3 × 15 s) (MSE Soniprep Tip Sonicator). After centrifugation (5 min, 3000 g), total RNA was extracted from the supernatant with an RNA extraction kit (PureLink™ RNA Mini Kit). RNA quality was evaluated by the A260/A280 ratio as measured with a Nanodrop spectrophotometer (NP80, Westburg). Two µg total RNA was reverse transcribed with the cDNA SuperMix (Quantabio, #95 048-100) in a total reaction volume of 20 µl using the PCR System 9700 (Applied Biosystems) according to the manufacturer’s instructions. For RT-qPCR, the Perfecta Sybr Green SuperMix (Quantabio, #95 054-500) was used. Reaction mixes were prepared in 10 µl volumes, containing 0.2 µl of the cDNA, according to the manufacturer’s instructions. RTqPCR was performed on a LightCycler^®^ 480 (Roche) with a hold stage of 2 min at 50°C followed by 2 min at 95°C. Next, 40 cycles of 15 s denaturation at 95°C and 45 s of primer annealing and extension at 60°C were used. All conditions were assessed in experimental replicates (*n* = 3–6) and each replicate was tested with technical triplicates by RT-qPCR. Threshold cycles were determined and used to calculate the expression fold changes within experimental conditions using the Pfaffl method.

**TABLE 1:** List of the primers used for RT-qPCR analysis.

Gene	Orientatio n	Primer Sequence (5′-3′)	Amplicon Size (bp)
MYH1	Forward	GGG AGA CCT AAA ATT GGC TCA A	106
	Reverse	TTG CAG ACC GCT CAT TTC AAA	
MYH3	Forward	CTT GTG GGC GGA GGT CTG G	119
	Reverse	AGC TAT GCC GAA CAC TTC CAT	
MYH8	Forward	ACA TTA CTG GCT GGC TGG AC	143
	Reverse	ACC TTT CTT CGC GCT GCT AT	
*References genes*			
GAPDH	Forward	TCA AGA AGG TGG TGA AGC AGG	168
	Reverse	ACC AGG AAA TGA GCT TGA CAA A	
HSP90AB1	Forward	AGA AAT TGC CCA ACT CAT GTC C	75
	Reverse	ATC AAC TCC CGA AGG AAA ATC TC	
RPL13A	Forward	CCT GGA GGA GAA GAG GAA AGA GA	126
	Reverse	TTG AGG ACC TCT GTG TAT TTG TCA A	

### Statistics

Statistical analysis was performed using GraphPad Prism software (version 8.0.2). Normality tests (Shapiro-Wilk) were performed on data before performing ANOVA tests. Unless described otherwise, non-parametric Kruskal–Wallis tests with Dunn’s post-tests were used when comparing the three culture conditions. For multiple comparisons, the mean rank of the stimulated conditions was compared only with the static culture. Biological triplicates were used, and at least 3 measurements were taken for each parameter reported. In the text, all data are reported as mean ± standard deviation and significance levels are indicated with *p < 0.05, **p < 0.01, and ***p < 0.001. Boxplot graphs show the data as the median with 5 and 95% percentiles.

## Results

### Determining the Effect of PEMF Stimulation on 2D Cultured Human Myoblasts During Myotube Formation

**FIGURE 2:**
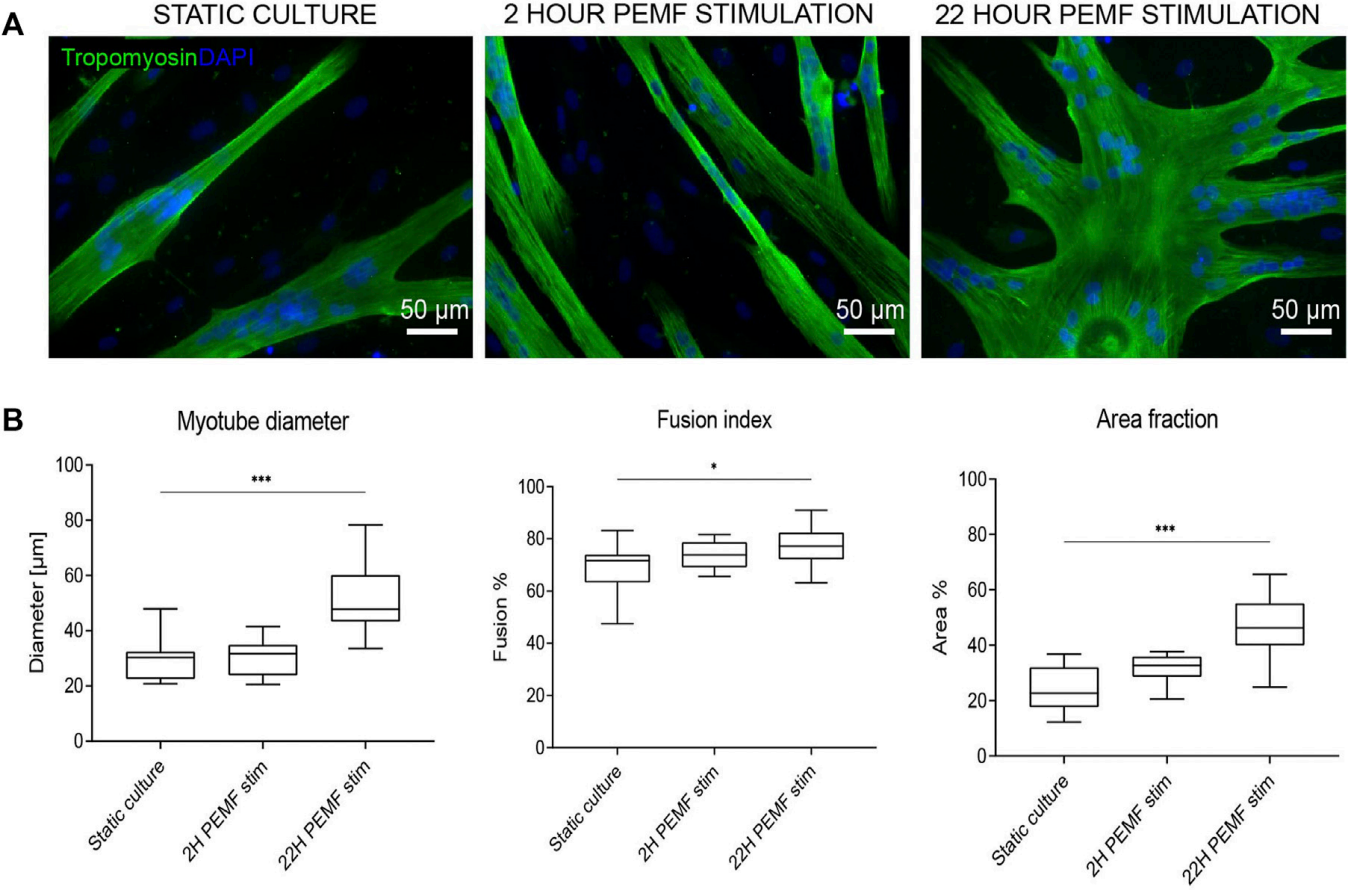
**(A)** Tropomyosin staining of 2D samples after no, 2 h per day or 22 h per day PEMF stimulation for 4 days. Blue: nuclei stained with DAPI; green: myotubes stained for tropomyosin. Scale bars: 50 µm. **(B)** Quantification from the tropomyosin staining images *via* Fiji software, diameter (left), fusion index (middle) and area fraction (% of surface covered by cells) (right) (*p < 0.05, **p < 0.01, ***p < 0.001) (n = 3 experimental replicates, for each 5 random images were analysed).

First, to determine whether the pulsed electromagnetic field stimulation can induce an enhanced formation of multinucleated myotubes, the primary human myoblasts seeded onto 24-well plates were stimulated for either 2 h or 22 h per day using the bioreactor setup as shown in Figure 1. These two different exposure times were applied starting at 24 h post cell seeding to allow for cell adhesion. Stimulation was maintained for 4 days of which 1 day in growth medium and 3 days in differentiation medium. After this period, myotube formation was assessed by quantification of the percentage of the surface occupied by tropomyosin positive myotubes. Representative images are shown in Figure 2A. Cultures stimulated for 22 h per day showed an overall significant increase in myotube formation (46 ± 10%) compared to statically cultured cells (24 ± 8%) as shown in Figure 2B. In addition, myotubes stimulated for 22 h were found to have a significant increase in diameter (51 ± 12 µm) compared to statically cultured myotubes (30 ± 7 µm). The fusion index was determined to estimate the fusion capacity of the cultured myoblasts within the different stimulation conditions. The fusion index was found to be slightly increased in the 22 h per day stimulation condition (77 ± 7%) compared to statically cultured myotubes (69 ± 9%) (Figures 2A,B).

### Optimal Duration of PEMF Stimulation During Myotube Formation in BioArtificial Muscles

**FIGURE 3:**
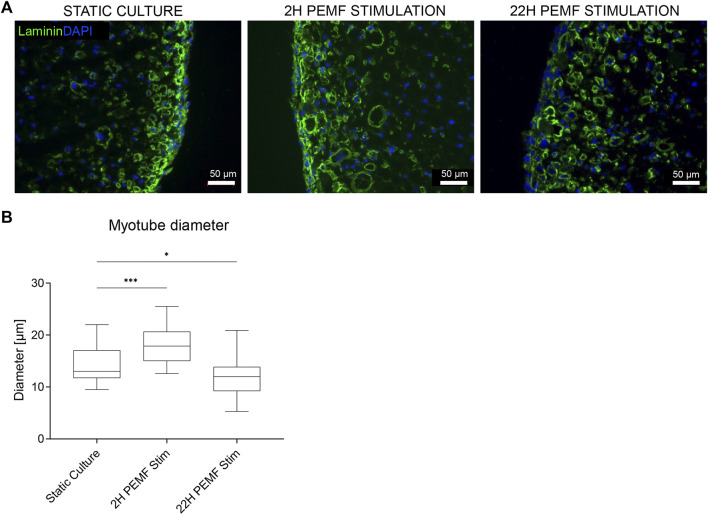
**(A)** Laminin staining of the 3D samples. Blue: nuclei stained with DAPI; green: myotubes stained for laminin. Scale bars: 50 µm. **(B)** Myotube width quantifications from the laminin staining images *via* Fiji software. (*n* = 6 BAMs, for each 3 images quantified, ***p < 0.001). The boxplot represents the median, 5 and 95% percentiles.

Since cellular behaviour is known to be different in a 3D environment versus monolayers on a plastic dish, we evaluated the impact of the previously applied PEMF stimulus durations on human muscle cell differentiation in a 3D culture format. Thus, human myoblasts were embedded within a fibrin hydrogel and cast in a custom silicone mold containing two attachment sites. The cells self-organize in the longitudinal direction due to cell-induced contraction of the hydrogel, resulting in a compact cylindrical-shaped 3D tissue by the end of the differentiation phase on day 8. Stimulation was carried out daily up to 16 days of the culture period, starting 24 h after casting. The samples were cultured for 3 days in the growth medium, 5 days in fusion medium and finally again 8 days in growth medium. Two hours of stimulation per day was effective in enhancing myotube cross-sectional diameter (18 ± 4 μm, *n* = 6), compared to the static culture (15 ± 4 μm, *n* = 6) (Figures 3A,B). On the other hand, differently in the 2D condition, the 22 h of stimulation/day resulted in a slight, but a nonsignificant reduction of myotube diameter (12 ± 3 μm, *n* = 6 *vs*. 15 ± 4 μm, *n* = 6) (Figures 3A,B).

### Determination of Optimal Time Window for PEMF Stimulation During 3D Myotube Formation

**FIGURE 4:**
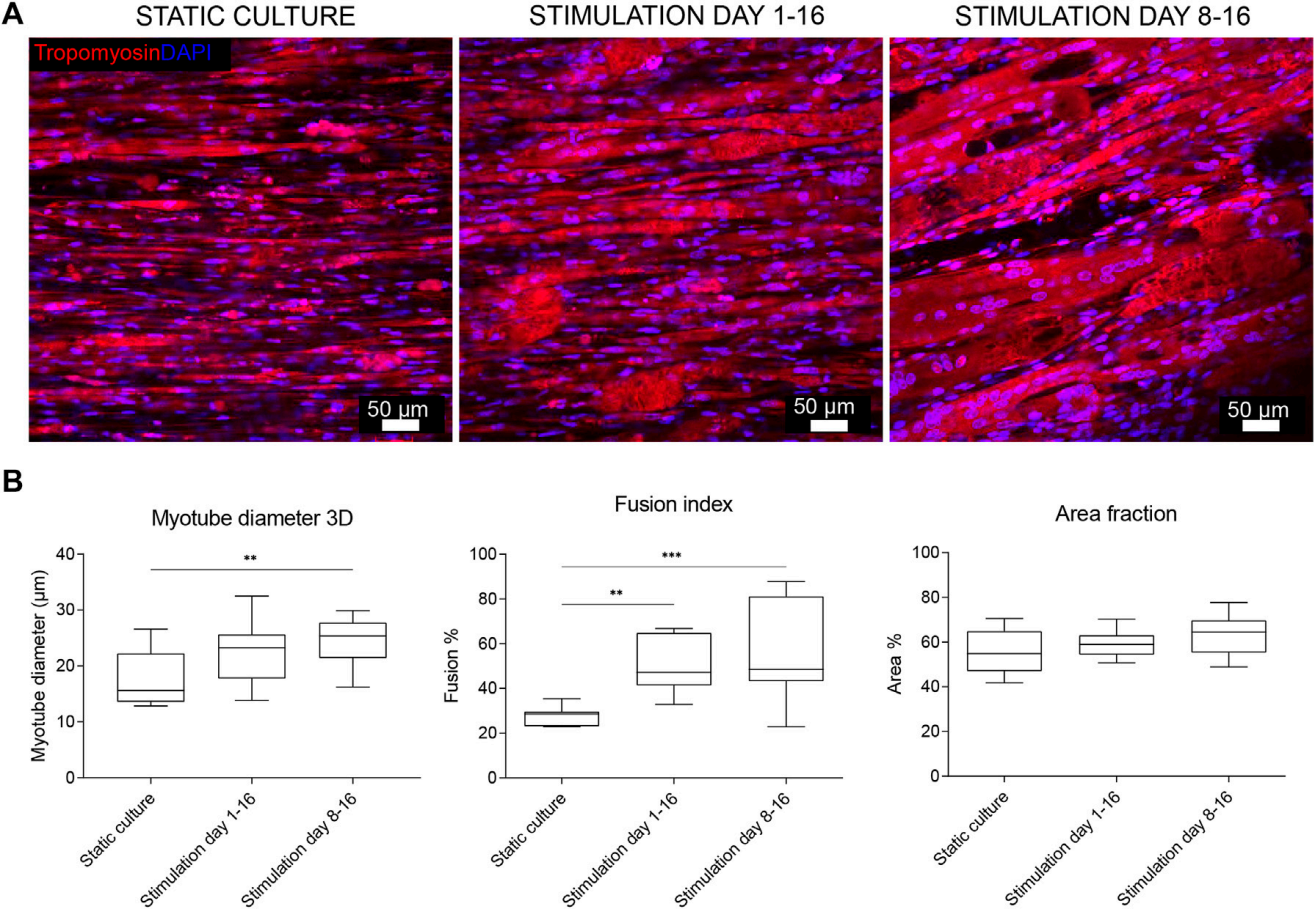
**(A)** Confocal microscopy images of BAMs not stimulated (static culture), PEMF stimulated for 2 h/day during the entire myotube formation (days 1–16) or only after 5 days of unstimulated myoblast fusion (PEMF stimulation days 8–16). Blue: nuclei stained with DAPI; red: myotubes stained for tropomyosin. Scale bar: 50 µm. **(B)** Quantifications from the tropomyosin staining images via Fiji software. (*n* = 2 control, *n* = 3 stimulated conditions).

**FIGURE 5:**
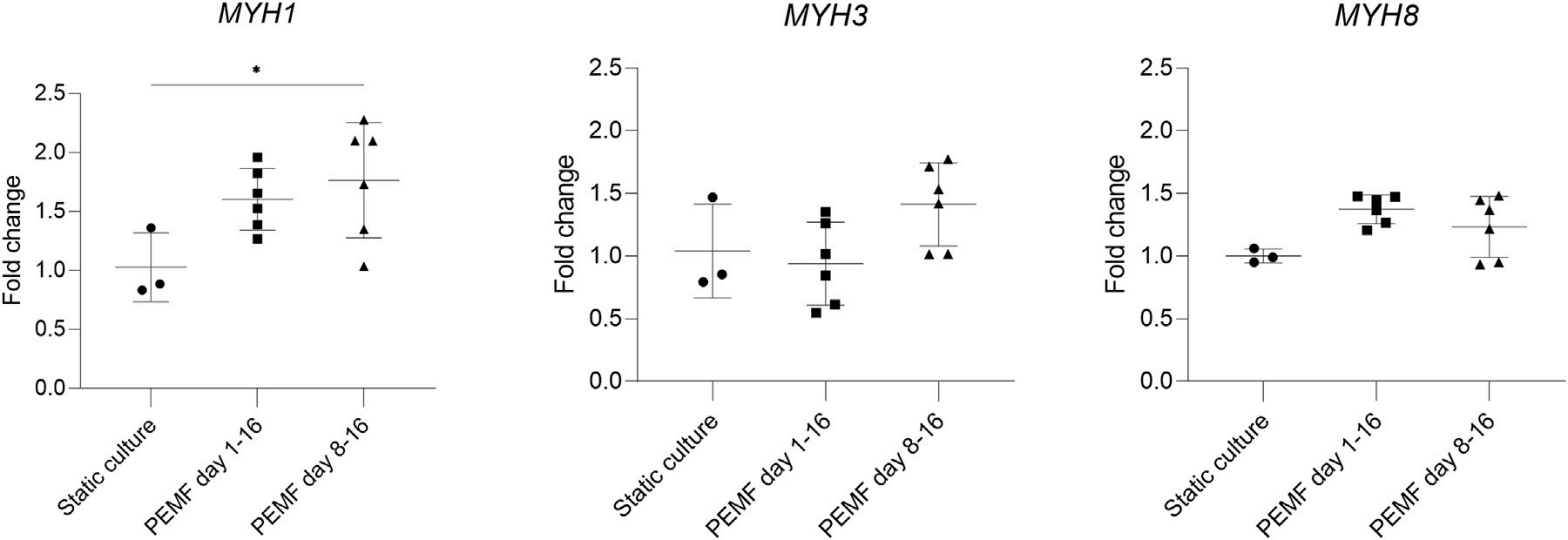
RT-qPCR analysis of the constructs (*n* = 3 control, *n* = 6 stimulated conditions). PEMF groups were compared to static culture by one-way ANOVA. The difference in expression of *MYH1* indicates a tendency for mature construct formation.

In previous experiments, stimulation was started early during the formation of myotubes, overlapping with the time window when cells still actively fuse. We next investigated whether this was a good starting point for applying the PEMF stimulation protocol on BAM constructs, or whether it would be preferential to allow cell fusion before the PEMF stimulation. Therefore, we compared static controls with two other groups where the stimulation was applied from day 1 until day 16 or from day 8 until day 16 (coinciding with switching back to a high serum growth medium). Overall, myotube formation was found to be improved with a significantly higher number of myoblasts fusing into myotubes for both stimulation conditions compared to the control or static culture (Figures 4A,B). More specifically, statically cultured 3D constructs had a fusion index of 28 ± 4%, while 3D constructs stimulated from day 1 until day 16 had a fusion index of 51 ± 11% and stimulating from day 8 until day 16 further improved the fusion index to 66 ± 18%. Myotube diameter increased as well when stimulation was applied between day 8 and 16 of culture (25 ± 15 μm, *n* = 3) and was found to be significantly higher compared to static culture (18 ± 9 μm, *n* = 2) but not significantly different from stimulation applied from day 1 onwards (23 ± 14 μm, *n* = 3). When checking the area fraction per image taken up by myotubes, an increasing trend for the stimulated conditions, but with no significant difference, was found. RT-qPCR was performed to assess possible differences in myosin heavy chain isoform expression. We quantified *MYH1* (adult fast IId), *MYH3* (embryonic) and *MYH8* (perinatal), determining which differentiation stage is enhanced. As shown in Figure 5, samples stimulated during the differentiation phase (stimulation applied from day 8 until day 16) show an increased *MYH1* expression (relative to the average of the reference genes) (1.8 ± 0.5, *n* = 6) when compared to the control condition (1.0 ± 0.3, *n* = 3). A similar, yet lower, tendency is observed when continuous stimulation is applied throughout the whole culture period (stimulation applied from day 0 until day 16) (1.6 ± 0.3, *n* = 6). Both PEMF stimulation conditions did not result in a significant difference in the expression of *MYH3* nor *MYH8*.

## Discussion

To enhance myoblast fusion and increase myotube diameter within engineered skeletal muscle, several types of biophysical stimuli have been studied. Mostly, mechanical stimulation (Powell et al., 2002; Boonen et al., 2010; Aguilar-Agon et al., 2019) and electrical stimulation (Langelaan et al., 2010; Khodabukus et al., 2015; Khodabukus et al., 2019), and the synergistic effects have been explored (Somers et al., 2017; Maleiner et al., 2018). Tissue-engineered skeletal muscle constructs are expected to highly benefit from biophysical stimulation due to their intrinsic adaptive nature to exercise. While PEMF devices are being sold for muscle pain relief, only a few papers have described the influence on skeletal muscle of patients (Kim et al., 2012; Jeon et al., 2015). Interestingly, 30 Hz mechanical vibrations have been suggested to induce muscle hypertrophy in newborn mice *in vivo* and enhance terminal differentiation of satellite cells *in vitro*. Furthermore, high-frequency stimuli (30-90 Hz) have been suggested for musculoskeletal regenerative rehabilitation therapy (Thompson et al., 2016; Jones et al., 2020) which may act through activation of a mechano-sensitive signalling pathway (Uzer et al., 2015). Finally, a systematic review including trials that evaluated the effect of localised vibration on muscle strength in healthy individuals concluded that vibrations (8-300 Hz) can enhance the strength in healthy adults (Alghadir et al., 2018). To our knowledge, however, no studies have described the influence of PEMF on tissue-engineered skeletal muscle development. In this work, we applied the human BAM model to study the effects of PEMF on myoblast fusion, myotube size and myosin heavy chain isoform expression.

The biophysical PEMF stimulation protocol was optimized to enhance the development of human tissue-engineered skeletal muscle. In the first explorative phase, the stimulation was applied on 2D cultured human myoblasts, seeded on gelatin-coated culture plates, to explore the effect of the electromagnetic stimulus. Two different exposure times were selected in this explorative phase: 2 and 22 h, based on what was previously observed on myocytes and osteoblasts (Fassina et al., 2006; Fassina et al., 2009; Ceccarelli et al., 2013). In fact, for the osteoblasts, 24 h of exposure to PEMF increased the extracellular matrix deposition (Fassina et al., 2006; Patterson et al., 2006; Fassina et al., 2009), while 1-h exposure enhanced the proliferation of myofibroblasts in a diabetic cutaneous wound healing model (Cheing et al., 2014) and 3 h enhanced differentiation of C2C12 cells (Liu et al., 2017). For our bioreactor setup, we chose two exposure durations, 2 *versus* 22 h of PEMF stimulation per day. The cells stimulated for 22 h showed a significant enhancement of myotube diameter, and an increased overall area occupied with tropomyosin positive cells in 2D. Nonetheless, the 2 h PEMF-exposed cells showed an increasing trend of these parameters in comparison to the static culture, albeit non-significant. For both stimulation durations, no difference was found in the myoblast fusion index, however, this may be because the human myoblasts we used already had a high fusion index (>70%) in the unstimulated control, leaving little room for further improvement. To further evaluate the effect of PEMF, we switched to a human 3D bio artificial muscle model which more closely mimics native skeletal muscle. The same PEMF daily stimulation durations ranging from 2 to 22 h were applied and evaluated. Two hours of stimulation was found to enhance the myotube formation within 3D cultured BAMs, demonstrated by a significant increase in myotube diameter. In contrast to the 2D results, a longer stimulation of 22 h per day resulted in a myotube diameter similar to unstimulated control. The different behaviour of cells in 3D is the reason that there is an increasing shift towards using 3D models. Cells experience a different mechanical environment and form other cell-cell and cell-matrix contacts. Therefore, cells have a different arrangement of cytoskeletal organisation which in turn influences signalling; this concept is called tensegrity and it was already described nearly three decades ago (Ingber, 1993; Mammoto and Ingber, 2010). Since the BAMs are based on an initial fibrin hydrogel that surrounds the myoblasts, the tension to which the cells are exposed is very different from adherence to tissue culture plastic. In addition, the value of resting is generally accepted in muscle physiology but not yet studied in the SMTE field. Similar to what is known in sports physiology, resting time is important to allow proper muscle development. Still, the value of resting is not agreed upon in the field and various durations are described. Our 3D model was able to capture a deleterious effects of 22 h stimulation. The model may in the future aid in further dissecting the mechanisms behind positive versus negative effects of stimulation duration.

Using the 2 h of stimulation as the best exposure duration, we questioned whether the outcome could vary with starting the stimulation at different time points during the BAM development process. In literature, different protocols are reported with different starting points in the culture period, but the general tendency is that once the stimulation begins, it is generally applied every day until the end of the experiment (Patterson et al., 2006; Ceccarelli et al., 2013; Cheing et al., 2014; Liu et al., 2017). Since no general guidelines are reported, we compared two different starting points, being stimulation early on before myoblast fusion to myotubes occurred versus starting stimulation only after myotube formation. Myotube formation is typically induced in serum starvation medium for 4-5 days. In our protocol, this fusion induction was from days 3–8. Stimulation from days 8–16 as well as stimulation from days 1–16 resulted in an overall improved myotube formation as compared to the static control. Applying the stimulation after the formation of the myotubes resulted in the largest improvement with myotubes having an increased diameter and a higher area of cells versus the extracellular matrix. Interestingly, the fusion index was further augmented, also in the group where PEMF stimulation only started after fusion for 5 days. This indicates that PEMF is efficient in further stimulating myoblast fusion even when the medium is already returned to a serum-rich growth medium. Further characterization on the molecular level by RT-qPCR analysis for the different myosin heavy chain isoforms showed that the chosen condition displayed an increased expression of the mature form of myosin heavy chain (*MYH1*), indicative of an enhanced myotube formation within the construct.

PEMF stimulation has not been applied thus far in the context of muscle tissue engineering. It was the purpose of this work to establish if this PEMF stimulation affected the early stages of differentiation. The presented findings are encouraging and warrant further follow-up studies in which many other parameters and ultimately more functional analysis can be performed. In this work, we focused on fixed PEMF stimulus to determine solely the impact of exposure duration and starting point. Still, a wide range of PEMF stimulation protocols can be set up by varying other field parameters such as frequency and magnitude. The current study had a well-delineated scope and served to open a novel subfield.

In summary, the present work has studied the use of PEMF stimulation for human skeletal muscle tissue engineering. We demonstrated that PEMF stimulation can enhance myoblast fusion and can increase myotube diameter, with myotubes expressing more of the adult myosin heavy chain isoform. We also show that the BAM model can be used to study the effects of muscle stimulation protocols and that the effects on this 3D model are different from what is observed in 2D.

## Data Availability

The datasets presented in this study can be found in online repositories. The names of the repository/repositories and accession number(s) can be found below: https://osf.io/59k3g/, 59k3g.
